# Toll-Like Receptor 3 Expression in Glia and Neurons Alters in Response to White Matter Injury in Preterm Infants

**DOI:** 10.1159/000346158

**Published:** 2013-03-16

**Authors:** R. Vontell, V. Supramaniam, C. Thornton, J. Wyatt-Ashmead, C. Mallard, P. Gressens, M. Rutherford, H. Hagberg

**Affiliations:** ^a^Centre for the Developing Brain, Division of Imaging Sciences and Biomedical Engineering, The Rayne Institute, King's College London St. Thomas' Hospital, London, UK; ^b^Perinatal Imaging Group, Centre for the Developing Brain, MRC Hammersmith Hospital, London, UK; ^c^Centre for the Developing Brain, Institute of Reproductive and Developmental Biology, Department of Surgery and Cancer, Imperial College London Hammersmith Hospital, London, UK; ^d^Wigglesworth Perinatal Pathology Services, St. Mary's Hospital, London, UK; ^e^Institute of Neuroscience and Physiology, Sahlgrenska Academy, Göteborg University, Göteborg, Sweden; ^f^Inserm, U676, Paris, France

**Keywords:** Toll-like receptor 3, White matter injury, Inflammation, Astrocytes, Neuronal proliferation

## Abstract

Toll-like receptors (TLRs) are members of the pattern recognition receptor family that detect components of foreign pathogens or endogenous molecules released in response to injury. Recent studies demonstrate that TLRs also have a functional role in regulating neuronal proliferation in the developing brain. This study investigated cellular expression of TLR3 using immunohistochemistry on human brain tissue. The tissue sections analysed contained anterior and lateral periventricular white matter from the frontal and parietal lobes in post-mortem neonatal cases with a postmenstrual age range of 23.6-31.4 weeks. In addition to preterm brains without overt pathology (control), preterm pathology cases with evidence of white matter injuries (WMI) were also examined. In order to identify TLR-positive cells, we utilized standard double-labelling immunofluorescence co-labelling techniques and confocal microscopy to compare co-expression of TLR3 with a neuronal marker (NeuN) or with glial markers (GFAP for astrocytes, Iba-1 for microglia and Olig2 for oligodendrocytes). We observed an increase in the neuronal (28 vs. 17%) and astroglial (38 vs. 21%) populations in the WMI group compared to controls in the anterior regions of the periventricular white matter in the frontal lobe. The increase in neurons and astrocytes in the WMI cases was associated with an increase in TLR3 immunoreactivity. This expression was significantly increased in the astroglia. The morphology of the TLR3 signal in the control cases was globular and restricted to the perinuclear region of the neurons and astrocytes, whilst in the cases of WMI, both neuronal, axonal and astroglial TLR3 expression was more diffuse (i.e., a different intracellular distribution) and could be detected along the extensions of the processes. This study demonstrates for the first time that neurons and glial cells in human neonatal periventricular white matter express TLR3 during development. The patterns of TLR3 expression were altered in the presence of WMI, which might influence normal developmental processes within the immature brain. Identifying changes in TLR3 expression during fetal development may be key to understanding the reduced volumes of grey matter and impaired cortical development seen in preterm infants.

## Introduction

Fetal neuronal and glial development is highly active between 8 and 32 weeks of gestation. During this time, there are proliferative regions of the brain, such as the germinal matrix, that are vulnerable to injury as seen in germinal matrix haemorrhage/intraventicular haemorrhage or viral infections such as cytomegalovirus. Neuronal migration from the germinal matrix to the cerebral cortex is almost complete by 24 weeks of gestation [1,2]. This process may be impaired in the very preterm infant who sustains a germinal matrix haemorrhage. Premature infants are also susceptible to periventricular white matter injury (WMI) between 22 and 32 weeks of gestation [1,3,4]. Injury in these regions is thought to be the result of inflammation/infection and ischaemia with immature oligodendrocytes showing increased susceptibility. WMI affects both local cell survival and maturation [5] and may inhibit axonal growth, cortical development and subsequent thalamic cortical connectivity occurring during this time period [6,7].

In human preterm infants, WMI is identified by grey sunken soft tissue patches seen macroscopically. Microscopically the histopathology of WMI shows dissolution of the white matter, granular neurophil, axonal injury [8,9] and foamy appearing histiocytes [10]. WMI is associated with a disturbance to differentiating premyelinating oligodendrocytes, impaired myelination, astrogliosis and microglial activation [11,12,13]. WMI has characteristic signs of focal cysts and diffuse low signal intensity in remaining white matter on both ultrasound and magnetic resonance imaging (MRI) during the neonatal period [14,15]. Despite advances in neonatal care, there is still significant mortality and morbidity arising from injuries to the developing brain following preterm delivery. Preterm infants have high rates of injury to white matter structures and subsequent reduced volume of grey matter structures [16,17,18].

In the innate immune system, toll-like receptors (TLRs) and other pattern recognition receptors recognize pathogen-derived compounds (pathogen-associated molecular patterns) or endogenous molecules released by the host in response to an infection or inflammatory injury (damage-associated molecular patterns) [19]. Pathogen-serum complexes activate macrophages and microglia that respond to the injury [20,21] via different pattern recognition receptors, such as TLRs [22]. For instance, TLR3 has been identified intracellularly in the endosomal membrane in a variety of cells and has been shown to detect viral molecular patterns [23], such as double-stranded RNA, associated with viral infection [24].

In recent years, TLRs were identified in the mammalian nervous system. Members of the TLR family were originally thought to be limited to the glial cells; however, recent in vitro studies suggest that neurons and neural progenitor cells express TLRs and regulate cell fate decision [25,26,27,28,29]. Several TLRs have been reported to be expressed in developing neurons and influence both proliferation and differentiation [26,27]. Recent studies demonstrate that TLR3 has a functional role in regulating neuronal proliferation in the developing brain [26,27,28,29]. TLR3 activation via synthetic polyinosinic-polycytidylic acid or mRNA has been shown to reduce the numbers of neurospheres as it is concentrated in the growth cone and can cause growth cone collapse inhibiting neurite extension [28]. Thus studies on the consequences of TLR3 activation are necessary for comprehending developmental and degenerative pathologies seen in the central nervous system after infection and/or damage.

The role of TLR3 and the cellular populations which express this protein in the developing human brain needs to be clarified. Our hypothesis is that in cases of WMI, there is a redistribution of TLR3 receptors in neurons and enhanced TLR3 expression in certain microglia and astroglial populations. An improved understanding of the expression of TLR3 will provide important insights into its role in the normal and abnormal developing brain and potentially inform on therapeutic strategies.

## Materials and Methods

Informed parental consent was acquired according to National Health Service UK guidelines. Study ethics were obtained from the National Research Ethics Service, Hammersmith and Queen Charlotte's and Chelsea Research Ethics Services, London, UK. Fourteen extremely preterm post-mortem brains [<32 weeks gestational age (GA)) of vaginally delivered neonates were obtained from the Perinatal Pathology Department, Imperial Health Care Trust, London, UK. The primary cause of death of each case was assessed by a pathologist (J.W.A.). Brain tissue blocks of these 14 cases stored in our neonatal brain bank were used in this study and had a postmenstrual age (PMA) range from 23.6 to 31.4 weeks determined by GA plus age at death (weeks). Amniotic fluid infections were identified in the majority of the cases; however, none of the cases had leptomeningitis or vascular thrombosis. Seven of the brains which showed no significant brain pathology on gross and microscopic examination from post-mortem examination and no visible brain abnormalities on post-mortem MRI were used as non-neuropathological controls (control cases). The remaining 7 brains showed focal lesions with evidence of WMI upon pathological examination (WMI cases). Three of the controls and 2 of the WMI cases showed evidence of focal necrotizing enterocolitis. Congestive heart failure was noted in 5 of the controls and in 3 of the WMI cases. Case details are shown in table 1.

### Tissue Preparation and MRI

The bodies were refrigerated (2-4°C) prior to post-mortem examination which was performed within 1-3 days of death. Whole post-mortem brains were fixed in 4% formalin for 5-7 weeks depending on size. Towards the end of the fixation period, MRI was performed on fixed whole brains at 3 Tesla. T1- and T2-weighted images were obtained in 3 orthogonal planes. Visual analysis was performed by an experienced neuroradiologist to confirm normal anatomy and absence of pathology in all control cases and the confirmation of WMI in the WMI cases. The whole brains were sliced by a pathologist (J.W.A.) and the tissue blocks were processed on a Bright Tissue Processor (Bright lnstrument Co. Ltd., Cambridgeshire, UK). Then the paraffin-embedded tissue blocks were sectioned at 6 μm using a Leica RM2245 microtome (Leica Microsystems Ltd., Bucks, UK). Paraffin-embedded tissue sections from the frontal and parietal lobes (at the level of Ammon's horn) were used for immunohistochemistry.

### Immunohistochemistry

After routine paraffin removal and rehydration, endogenous peroxidase activity was quenched by placing the slides into 3% hydrogen peroxide (H_2_O_2_) for 10 min. Sections were immersed in preheated 10 mM citric acid (VWR International Ltd., Leicestershire, UK), pH 6.0, for 30 min and cooled in cold water. Sections were blocked in 5% goat serum (Vector Laboratories, Burlingame, Calif., USA) for 20 min before being incubated overnight at 4°C in a solution of rabbit anti-TLR3 (0.1 μg/ml; Abcam, Cambridge, UK) in PBS. Specificity of the antibody was determined by using synthesized antigenic peptides from the protein of interest in a 10-fold excess with respect to the primary antibodies. The next day, sections were exposed to the biotinylated goat-anti-rabbit IgG secondary antibody (15 μg/ml; Vector Laboratories) in PBS for 1 h followed by avidin-biotin complex for 1 h (1:200, ABC; Vector Laboratories). The reactions were visualized with 3,3′-diaminobenzidine (Sigma-Aldrich Company Ltd., Gillingham Dorset, UK) for 10 min. Finally, the sections were dehydrated, cleared in xylene and coverslipped. As negative controls, we performed staining in the absence of the primary antibodies and preabsorption with the TLR3 peptide (1.0 μg/ml; Abcam); no specific staining was identified in these preparations.

### Immunofluorescence Double-Labelling

To identify the cellular location of the TLR3 protein in our tissue immunofluorescence (IF) double-labelling was performed on the WMI and on the control cases. The mouse monoclonal primary antibodies that were compatible with the rabbit anti-TLR3 (0.1 μg/ml) were mixed in separate cocktail solutions in conjunction with the anti-TLR3, as follows: mouse monoclonal glial fibrillary acidic protein, clone G-A-5 (GFAP; 1:1,000; Sigma-Aldrich Company Ltd.), a marker for astrocytes, and oligodendrocyte lineage marker, clone 211F1.1 (Olig2; 1 μg/ml; Millipore, Temecula, Calif., USA). Sections were pretreated as described above and blocked in 5% goat serum for 20 min before the primary antibodies were applied and incubated overnight at 4°C. Following primary antibody incubation, sections were rinsed three times in PBS, for 3 min each time before these secondary antibodies were added. The samples were finally soaked for 1.5 h in PBS containing the following secondary antibody cocktail: goat anti-mouse IgG conjugated to Alexa Fluor 488 (4 μg/ml; Invitrogen, Eugene, Oreg., USA) and goat anti-rabbit IgG conjugated to Alexa Fluor 546 (4 μg/ml; Invitrogen).

In order to co-label with incompatible markers, such as a specific marker of microglia, rabbit anti-ionized calcium-binding adaptor molecule 1 (Iba-1; Wako Chemicals GmbH, Neuss, Germany), the antibodies were applied separately. Thus, after the sections were blocked in 5% goat serum for 20 min, the anti-TLR3 (0.1 μg/ml; Abcam) was applied and incubated overnight at 4°C. The sections were rinsed three times in PBS, for 3 min each time and were soaked for 1.5 h in PBS containing goat anti-rabbit IgG conjugated to Alexa Fluor 488 (4 μg/ml; Invitrogen) followed by 3 rinses in PBS. Additional block in 5% goat serum for 20 min was applied to the sections. During the blocking step, the Fab-antibody complex was made, 0.5 μg/ml of rabbit-anti-Iba-1 was conjugated to 2.0 μg/ml of Zenon Alexa Fluor 488 rabbit IgG (Invitrogen) for 5 min followed by 2.0 μg/ml of Zenon blocking reagent for an additional 5 min. The sections were then incubated in the Fab-antibody complex for 1.5 h, which was followed by PBS rinses and postfixation in 4% paraformaldehyde for 20 min. Another incompatible marker was rabbit anti-neuronal nuclear antigen (NeuN, Cy3 conjugate; Millipore), which stains the cytoplasm and nucleus of neurons. Identification of anti-TLR3 was the same as previously described in the TLR3/Iba-1 section, except the secondary antibody to detect TLR3 consisted of goat anti-rabbit IgG conjugated to Alexa Fluor 546 (4 μg/ml; Invitrogen). Following the identification of TLR3, the sections were rinsed in PBS and postfixed in 4% paraformaldehyde for 10 min followed by incubation with NeuN, Cy3 conjugate (1:500) for 1 h. Finally, the sections were placed under a coverslip using ProLong Gold antifade reagent with DAPI (Invitrogen) and then kept in the dark at 4°C until analysis.

### Microscopy

**Fig. 1:**
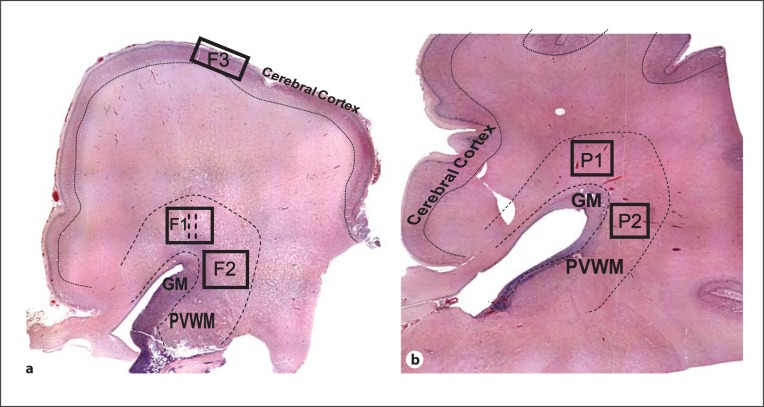
Global view of the frontal cortex (**a**) and the parietal cortex (**b**). The strategy for the quantification of the cell population and the TLR3-positive expression are demonstrated in WMI 1 (at 29.6 weeks PMA; **a**) and in WMI 2 (at 31.4 weeks PMA; **b**). Boxes represent the contours that were made in periventricular white matter regions of the frontal (F1, F2) and the parietal (P1, P2) lobes above the anterior germinal matrix (F1, P1) and adjacent to the lateral germinal matrix (F2, P2). The semi-quantitative analyses were completed in the periventricular white matter of the frontal lobe (dotted lines in F1). Cortical density was analysed in the anterior frontal lobe (F3). GM = Germinal matrix; PVWM = periventricular white matter.

An unbiased count of the number of TLR3-immunopositive cells and the total number of cells were screened by 2 individuals (R.V. and C.T.; blinded to the case data) using the optical fractionator program (MicroBrightfield, Inc., Colchester, Vt., USA). The sections were examined under bright-field microscopy using a light microscope (DM6000 B; Leica Microsystems Ltd., Bucks, UK) equipped with a motorized specimen stage for automated sampling (MicroBrightfield, Inc.), CCD colour video camera (MicroBrightfield, Inc.) and stereology software (Stereo Investigator, v8.27; MicroBrightfield, Inc.). The average area of each contour was 14 × 10^3^ mm^2^, which was made using a ×1.25 objective. Cells were counted in a 0.9 mm^2^ counting frame on an average of 54 counting sites and conducted using a ×40 objective. In the pilot study, the dissector profile described above estimated a sufficient number of cells and the coefficient of error (Schmitz-Hof) was within an acceptable range [30]. The cellular density and the TLR3-immunopositive cell counts were performed in the anterior (F1, P1) and lateral (F2, P2) regions of the periventricular white matter in the frontal and parietal lobes and in the frontal cortex (F3) as outlined using a standard haematoxylin and eosin stain (fig. 1).

Data were analysed with a Student t test or one-way ANOVA followed by Tukey's range test to compare the ratio of TLR3-positive cells between groups (WMI and control) in the periventricular white matter region of the frontal lobe and frontal-parietal lobe. Data were presented as mean ± SD and significance was set at p < 0.05. All statistical analyses were performed using GraphPad Prism 5.0 (GraphPad Software, San Diego, Calif., USA).

Multichannel images were captured with a Leica microscope DM6000 B (Leica Microsystems Ltd.) and processed in Image J (version 1.42; National Institute of Health, Bethesda, Md., USA) before final processing in Adobe Photoshope (version 11.0.2; Adobe Systems Inc., San Jose, Calif., USA). A semi-quantitative analysis through the centre of the contour of region F1 was used to assess the density of TLR3-positive neurons (NeuN) and astrocytes (GFAP). The average number of immunopositive cells was calculated from images taken with a ×40 objective (0.0426 mm^2^) and was counted using Image J (version 1.42) by 2 student volunteers (M.H.A. and C.S.A.) who were blinded to the case data. Confocal double-labelled microphotographs were captured using a Leica SP5 spectral confocal microscope with settings appropriate to the fluorophores present.

## Results

These experiments were designed to determine the distribution of TLR3 in periventricular white matter of preterm human brains (23-32 weeks of PMA) from the anterior frontal, lateral frontal, anterior parietal and lateral parietal regions (fig. 1). We also sought to determine whether the presence of WMI in the human brain altered the expression of TLR3. Furthermore, we investigated whether glial TLR3 expression differed between control cases and those with WMI, with the use of immunohistochemistry and IF techniques. In the control group, the causes of death were complications due to extreme preterm birth, pneumonia and congestive heart failure and the infant survival rate was less than 24 h. The WMI group had similar complications as the control group but lived up to 5 weeks longer than the control. Although the PMA of the WMI group was significantly higher than that of the controls (p < 0.05; WMI 28.86 ± 0.7978 weeks, n = 7; control 25.16 ± 0.6213 weeks, n = 7), these groups were not significantly different at birth (GA; p > 0.05). Case details are shown in table 1.

### The Ratio of TLR3-Positive Cells Increased in the Periventricular White Matter in WMI Cases

**Fig. 2:**
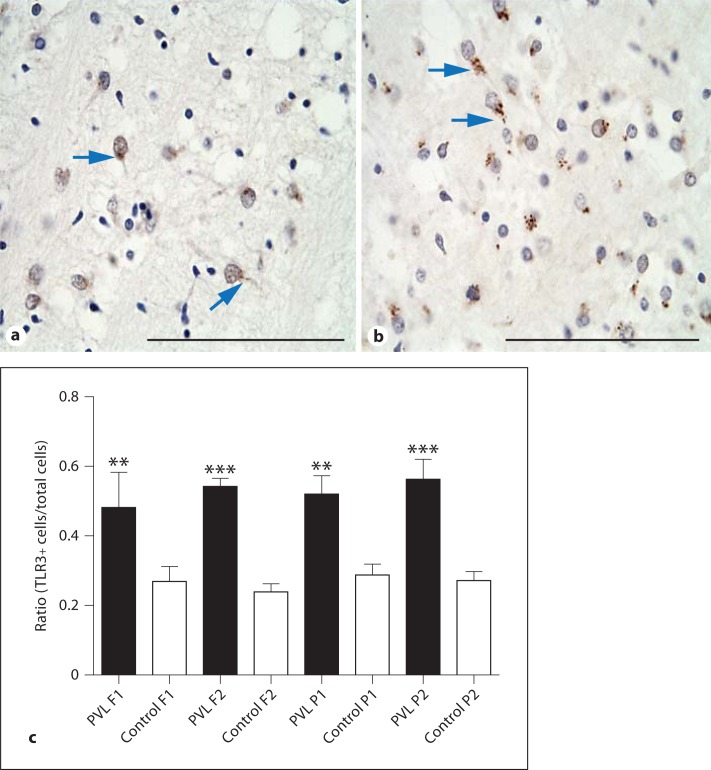
Immunohistochemical stains of TLR3 immunoreactivity in control 3 (**a**) and in the WMI case 2 (**b**) from the anterior region of the periventricular white matter (F1). In the first image (**a**; arrows), the immunoreactivity appears perinuclear and punctate, whereas in the second image (**b**; arrows), it is diffuse (i.e., less concentrated around the nucleus and flowing into the axon hillock). Scale bar = 100 µm. **c** A graph representing the ratio of TLR3-positive cells to the total cellular count. The asterisks indicate significant differences in the ratio of immunopositivity counted in the WMI cases compared to the control, in the anterior region of the periventricular white matter from the frontal (F1) and parietal (P1) lobes, and in the lateral region of the periventricular white matter from the frontal (F2) and parietal (P2) lobes. ** p < 0.01; *** p < 0.001.

In the periventricular white matter of the cases without overt brain pathology, the TLR3 immunoreactivity was globular and restricted to the perinuclear region of cells, which is consistent with endosomal staining (fig. 2a). The WMI cases also showed TLR3 expression in the periventricular white matter, but there was more diffuse staining outside of the nucleus of cells and within the processes in contrast to the more focal nuclear localization seen in non-injured brains (fig. 2b). One-way ANOVA showed a significant increase in cells with TLR3+ immunoreactivity in the WMI cases compared to the controls in the anterior frontal region (F1; p < 0.01; WMI 0.48 ± 0.11, n = 7; control 0.27 ± 0.12, n = 7) and in the lateral frontal region (F2; p < 0.001; WMI 0.54 ± 0.06, n = 7; control 0.24 ± 0.06, n = 7) (fig. 2c). A significant increase in cells expressing TLR3 in the WMI cases compared to controls was also found in both regions of the parietal lobe studied (P1 p < 0.01; WMI 0.52 ± 0.14, n = 7; control 0.25 ± 0.09, n = 7; P2 p < 0.001; WMI 0.56 ± 0.15, n = 7; control 0.27 ± 0.06, n = 7) (fig. 2c).

### TLR3 Immunolabelling in Neurons and Glial Cells

**Fig. 3:**
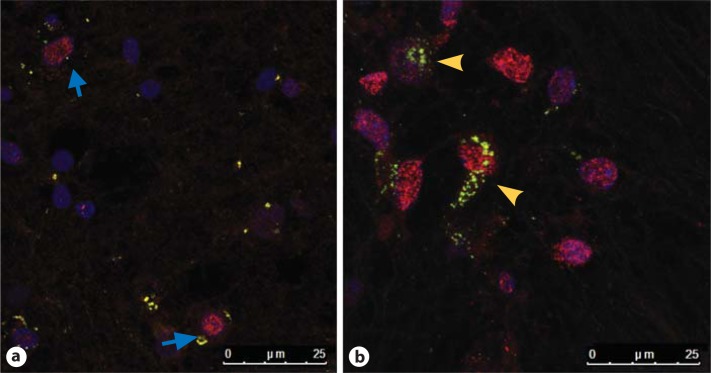
Confocal microscope photomicrographs demonstrating the distribution of TLR3 (green) around the nuclei (NeuN-Cy3) from the anterior region of the periventricular white matter in the frontal lobe of control case 5 (**a**) and WMI case 5 (**b**). Note that in the control case, TLR3 is located densely around the nucleus (blue arrows), whereas in the WMI case, the TLR3 expression is both nuclear and axonal (yellow arrowheads). Scale bar = 25 µm.

Of interest, the number of neurons identified with anti-NeuN was significantly higher in the WMI group (WMI 62.17 ± 11.20, n = 6; control 32.45 ± 2.259, n = 6; p < 0.01) than the control group in the region sampled (centre of F1). The amount of identifiable TLR3 expression seen in the neurons was not significantly different between the control and WMI groups, but to study differences in the pattern of neuronal expression, we examined the intracellular location of TLR3 protein by double-labelling IF techniques using rabbit-anti TLR3 and rabbit-anti-NeuN conjugated Cy3. Interestingly, in the control cases, the TLR3 expression was globular and restricted to the perinuclear region (fig. 3a), whereas in the WMI cases, the TLR3 immunoreactivity was diffuse and intra-axonal, being expressed along the axon hillock (fig. 2b, 3b).

To address whether the increase in TLR3 positivity seen in the periventricular white matter of the WMI brains was due to TLR3 activation in glial cells, IF colocalization was used to identify the glial population(s) that also express TLR3 protein within the frontal lobe. This was achieved by combining anti-TLR3 with glial markers, such as anti-GFAP (astrocytes), anti-Iba-1 (microglia) and anti-Olig2 (oligodendrocytes) in brain tissue sections.

**Fig. 4:**
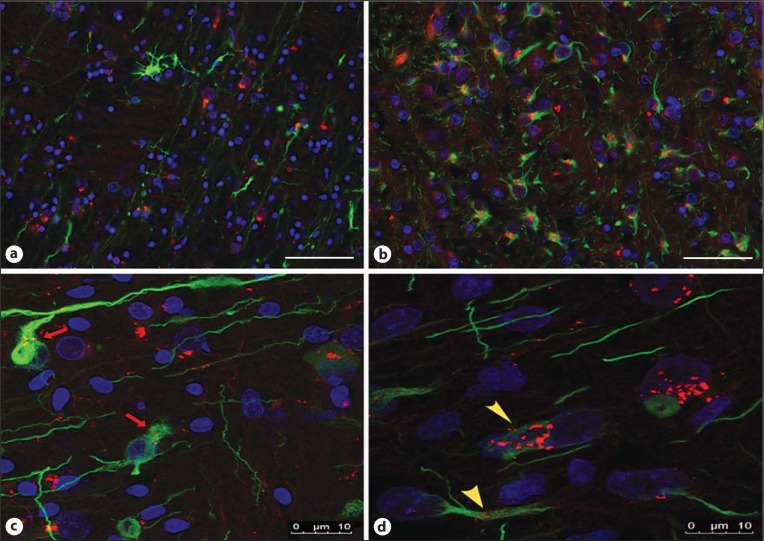
Examples of the IF staining of anti-TLR3 (red) and the astrocytic marker (GFAP; green) in one of the control cases (7; **a**, **c**) and in one of the WMI cases (1; **b**, **d**). In **b** and **d**, the astrocytes are reactive and prominent intracellular expression of TLR3 can be seen in the IF image and with the confocal photomicrograph (yellow arrowheads), whereas in **a** and **c**, there is less TLR3 expression in the astrocytes, which is confirmed by the confocal photomicrograph (**c**; red arrow). Scale bar = 50 µm (**a**, **b**) and 10 µm (**c**, **d**).

There was a significant increase in the number of astrocytes identified in the WMI group (p < 0.01; WMI 46.37 ± 4.309, n = 6; control 23.84 ± 2.151, n = 7). Double-labelling IF showed that astrocytes were the glial cells that predominately expressed TLR3, which significantly increased in the WMI group (WMI 31.24 ± 1.794, n = 6; control 16.56 ± 2.273, n = 7; p < 0.001). TLR3 expression was frequently seen along the processes of the reactive astrocytes outside of their irregularly shaped nuclei in the WMI cases (fig. 4b, d), whereas TLR3 was sparsely identified within astroglial processes in the control cases (fig. 4a, c).

**Fig. 5:**
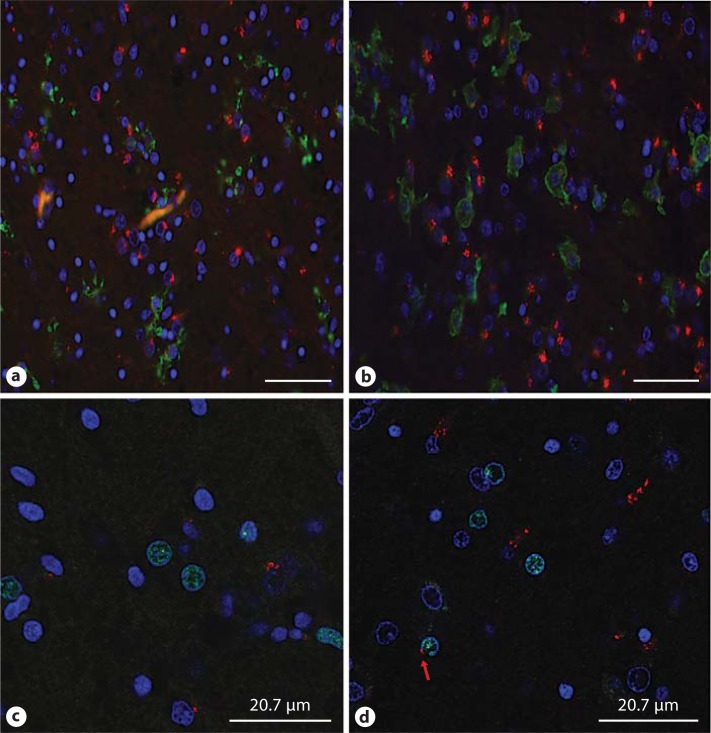
Double-labelling of anti-TLR3 (red) with the microglial marker Iba-1 (**a**, **b**; green) and the oligodendrogial marker Olig2 (**c**, **d**; green). The expression of TLR3 was not seen in the different morphologies of microglia, such as intermediate (control case 7; **a**) and amoeboidal (WMI case 1; **b**). Confocal microscopy confirmed that TLR3 expression was detected with Olig2 in the WMI case 7 (red arrow; **d**) but not in the control case 6 (**c**). Scale bar = 50 µm (**a**, **b**) and 20.7 µm (**c**, **d**).

Some TLR3 was identified adjacent to the nuclei of oligodendrocytes (i.e., those positive for Olig2), and this was more frequent in the cases of WMI. Identified in figure 5d in the photomicrograph is one Olig2 immunolabelled cell with TLR3 immunoreactivity seen in proximity to the nuclear membrane (fig. 5c, d). Surprisingly, similar low levels of TLR3 expression were seen within the cellular boundaries in microglia as determined by Iba-1 immunoreactivity. However, of interest, figure 5a and b demonstrates microglia of different morphologies located next to neurons with strong TLR3 expression, which may indicate an interaction between microglia and neurons via this receptor (fig. 5a, b).

### Fewer Cells in the Frontal Cortex in the WMI Group than in the Control Group

The number of neurons in the region F1 was higher in the WMI cases than in controls. In order to ascertain if this difference reflects a decrease in cortical density, we counted the cells in an average of 52 sites in a region of the superior frontal cortex (F3). In the control group, the cell density of the cortex was significantly higher in the region sampled of the frontal cortex compared to the WMI group (p < 0.05; control 1,455 ± 75.62, n = 7; WMI 1,012 ± 97.84, n = 6).

## Discussion

In this study, we have demonstrated the expression of TLR3 in several cell types of the preterm neonatal human brain; to the best of our knowledge, this is the first time this family of receptors has been demonstrated in the human neonatal brain. In addition, we showed that TLR3 expression was altered in the presence of overt brain injury, which was evident in the WMI cases compared with the controls.

The GA at birth was similar in the WMI and control groups, but survival was longer in the WMI group. We cannot exclude the possibility that WMI would have developed also in some control cases if they lived longer. Nevertheless, our data suggest that changes in TLR3 expression in neurons and glia accompanied development of WMI in the central nervous system of the extremely preterm infant.

TLR3 was detected in human brains that did not have WMI, which indicates that protein expression of TLR3 may be normal in fetal brain development even though we cannot exclude the possibility that TLR3 expression in the control brains is part of an early evolving pathological process related to extreme prematurity. In the preterm WMI cases, we saw more neuronal nuclei in periventricular white matter compared with the control cases. Whilst the overall expression within neurons was not increased, the pattern of expression of TLR3 was altered in WMI cases, being more diffuse and visible in the axonal hillock that extended into intracellular spaces of the neurons rather than being close to the endosome-like structures.

We hypothesized that in cases of neonatal injury, there would be an increase in microglia and astroglia populations, and that these would express TLR3. The periventricular white matter of the WMI group showed a wide spread of cellular populations that were positive for TLR3. Of interest is that this expression was predominantly on astroglia and not microglia as identified by double-labelling techniques.

It is useful to review the recent TLR3 animal model publications to further understand the significance of our findings and specifically to ascertain how overexpression may hinder the normal developmental process. Lathia et al. [27] found TLR3 expressed on glial cells, neurons and neural progenitor cells and polyinosinic-polycytidylic acid (an activator of TLR3) administration inhibited neurite extension in wild-type mice but not in TLR3 knock-out mice. Furthermore, Bsibsi et al. [28] found that a non-infectious microtubule regulator, stathmin, acted as an agonist for TLR3. During the inflammatory process of multiple sclerosis, TLR3 and stathmin were colocalized on the surface of astrocytes, neurons and microglia. In inflammatory injury of the preterm brain, such as WMI, there is a known increase in activated microglia [31] and astrocytes [16], which may underlie the documented alterations in neuronal connectivity between the thalamus and the cortex. In animal models of injury, stathmin or cytokines released from astroglia activate TLR3 in neurons of the premature brain, which in turn induces premature apoptosis [26,28,32].

Excessive TLR3 stimulation on astroglial cells may therefore interrupt the neuronal migration to the cortex. The increase in neuronal numbers seen in periventricular white matter and the decrease in the cerebral cortex in the WMI group and noted in other studies might indicate interference with the migration of late-migrating interneurons [8]. This may underly the alterations in thalamocortical connectivity and results in thalamic atrophy and/or cortical disorganization seen in WMI [33].

We have demonstrated the presence of TLR3 in all cell types of the immature brain. In the presence of WMI, TLR3 expression was increased and the pattern of expression altered. We speculate that this may play a role in the altered thalamocortical connectivity seen in preterm infants. The anatomical information gathered from our study provides a solid foundation for future studies of the axonal development, which is imperative in comprehending lifelong disabilities after premature birth.

## Figures and Tables

**Table 1:** Case data

Group	Sex	GA at birth weeks	Postnatal age	PMA at death weeks	Clinical context
Controls					
1	M	25.4	21 h	25.6	Respiratory distress syndrome and congestive heart failure as a result of extreme prematurity
	
2	M	23.86	9 h	23.9	Congestive heart failure and neonatal death due to extreme prematurity
	
3	M	26.3	1 d	26.4	Asymmetric intra-uterine growth restriction; neonatal death due to congestive heart failure
	
4	F	24.1	4 h	24.1	Confirmed amniotic fluid infection; neonatal death due to extreme prematurity
	
5	M	24.2	<1 h	24.2	Confirmed amniotic fluid infection; neonatal death due to extreme prematurity
	
6	M	23.6	<1 h	23.6	Prematurity, confirmed amniotic fluid infection, congestive heart failure
	
7	M	28.2	<1 h	28.2	Oligohydramnios; neonatal death due to extreme prematurity and congestive heart failure

Mean		26.8		26.9	

WMI cases					
1	M	27.7	12 d	29.6	Amniotic fluid infection, focal WMI, neonatal death due to congestive heart failure
	
2	F	28.9	18 d	31.4	Focal small GMH, focal WMI
	
3	F	27.7	22 d	30.6	Extensive WMI, congestive heart failure
	
4	F	27	7 d	28	Focal necrotizing enterocolitis, WMI, neonatal death due to extreme prematurity
	
5	M	24	5 wk 1 d	29.1	Focal necrotizing enterocolitis; confirmed amniotic fluid infection, WMI, death due to extreme prematurity
	
6	M	24.9	16 d	26.9	Mild oligohydramnios, confirmed amniotic fluid infection, early neonatal death due to congestive heart failure, WMI
	
7	M	25.7	1 d	25.9	Asymmetric intra-uterine growth restriction, WMI; neonatal death due to extreme prematurity

Mean		26.7		27.75	

GMH = Germinal matrix haemorrhage; M = male; F = female; d = days; wk = weeks; h = hours.
